# Assessment of seasonality of influenza in swine using field submissions to a diagnostic laboratory in Ontario between 2007 and 2012

**DOI:** 10.1111/irv.12248

**Published:** 2014-04-11

**Authors:** Zvonimir Poljak, Susy Carman, Beverly McEwen

**Affiliations:** a Department of Population Medicine, Ontario Veterinary College, University of GuelphGuelph, ON, Canada; b Animal Health Laboratory, University of GuelphGuelph, ON, Canada

**Keywords:** Diagnostic, influenza, monitoring, seasonality, swine

## Abstract

**Background:**

Seasonality of any infectious disease is important for its control and monitoring. While influenza seasonality in people has been evaluated extensively, this question has not been studied well in swine populations.

**Objective:**

The goal of this study was to investigate seasonality of influenza in swine, using diagnostic submissions to a diagnostic laboratory.

**Methods:**

Two thousand seven hundred and eleven virological tests within 685 submissions and 5471 serological tests within 193 submissions in Ontario swine between 2007 and 2012 were included in the study and converted to total monthly number of virological and serological submissions, and the number of positive submissions. Data were analyzed by time-series decomposition, fixed-effect Poisson, random-effect Poisson regression with month as uncorrelated and correlated random effects.

**Results:**

All approaches identified seasonality in virological submissions (*P* < 0·02) with peak in January and April, and a trough in July, but were not able to detect seasonality of influenza-positive virological submissions (*P* > 0·13). Seasonality of positive serological submissions was identified only if independence between months was assumed (*P* < 0·03). Almost 50% of serological submissions had evidence of exposure to H3N2 and H1N1.

**Conclusions:**

Thus, this study identified evidence of seasonality in influenza-like disease in swine herds, but not in circulation of influenza virus. Evidence of seasonality in exposure to influenza was dependent on assumptions of between-month correlation. High exposure to H3N2 and H1N1 subtypes warrants more detailed investigation of within-herd influenza virus circulation. The study provides initial insight into seasonality of influenza in swine and should be followed with herd-level studies.

## Introduction

Seasonality of any infection has profound implications for its transmission, and ultimately for monitoring and control programs. In human health, seasonality of many diseases is well studied, particularly influenza. It is generally accepted that in temperate regions, seasonal influenza in people peaks in the winter months^1^,^2^ although causes of such seasonality are still not completely understood.^3^–^6^ In contrast to human populations, existence of influenza seasonality in swine populations is not well studied. A general consensus among experts seems to be that swine influenza in the northern hemisphere has historically been a disease that peaked in the fall and early winter months, but that such seasonal pattern diminished with the advent of modern swine production systems.^7^ Nonetheless, little information based on quantitative data is currently available to describe a pattern of influenza in swine over different seasons. One study that explicitly addressed influenza seasonality in pigs was carried out recently in four European countries between 2006 and 2008.^8^ No evidence of between-season differences in proportion of influenza-exposure-positive pigs or farms could be found based on sampling that was restricted to summer and winter periods only. Seasonality of influenza has also been studied using diagnostic data from monitoring systems.^2^,^9^ A recent study from Minnesota used such an approach in swine populations.^10^ It therefore would be of interest to study influenza seasonality in Ontario swine using a similar approach where such data exist. In Ontario, type A influenza in swine is a common endemic infection, and veterinary practitioners regularly submit samples to diagnostic laboratories in order to investigate clinical problems suggestive of infection with influenza virus. The objective of this study is to investigate the existence of seasonality of influenza in Ontario swine based on diagnostic submissions to the largest Ontario Animal Health Laboratory between May 2007 and December 2012.

## Materials and methods

### Laboratory submissions and data processing

**Figure 1:**
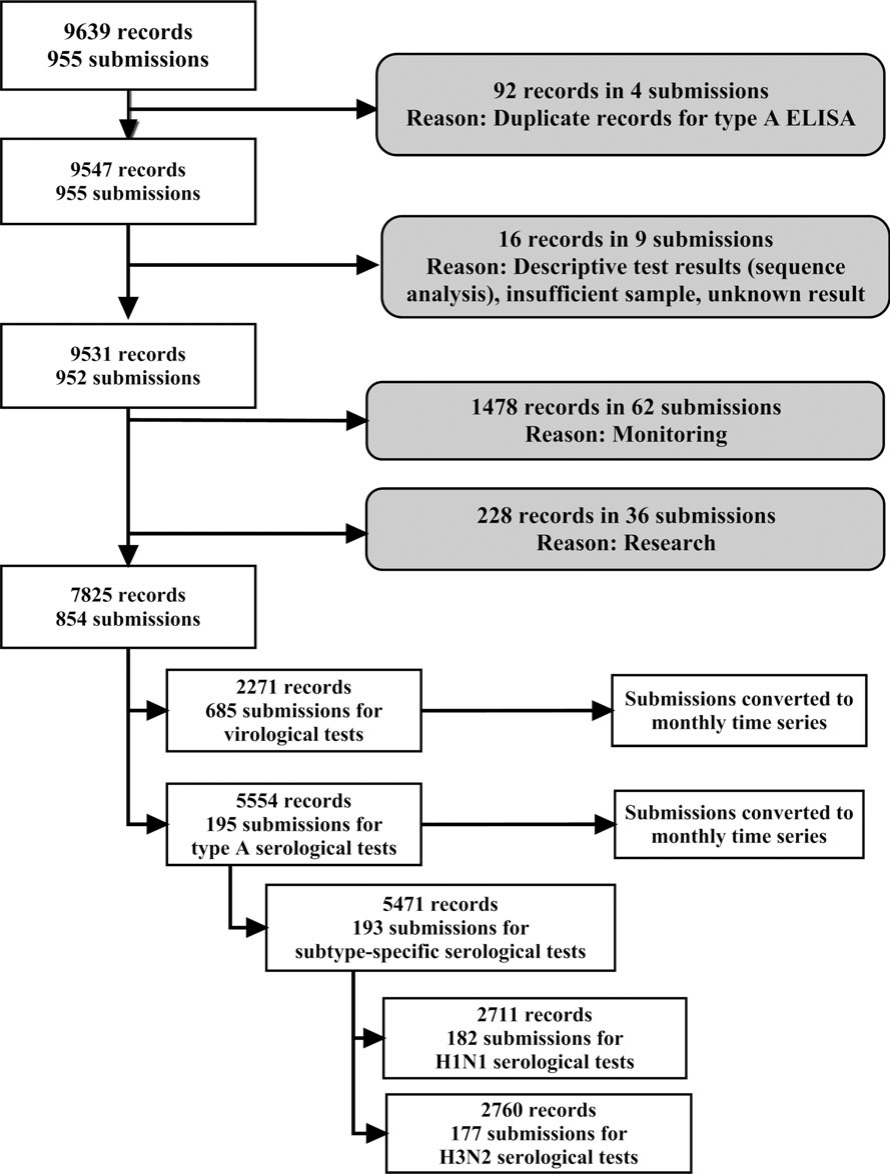
Flow of data in the study. Gray boxes indicate number of records that were excluded from the analysis, with the reason for exclusion.

Data on submissions for diagnostic tests for influenza in swine in Ontario between May 2007 and December 2012 were received from the Animal Health Laboratory (AHL, University of Guelph, Guelph, Ontario, Canada). May of 2007 was the month when a new data management system was implemented in the laboratory. The data received consisted of 9639 records, equivalent to individual test results, within 955 unique submissions (Figure1). Of these records, 3460 individual samples within 254 submissions were tested with more than one test. Ninety-two records were the results of the same test performed on the same sample but were reported on different scales, and they were excluded from analysis (Figure1). Results of tests submitted as a part of monitoring and research submissions were also excluded from the analysis, with a rationale that monitoring and research submissions might not be motivated by influenza-like disease in a herd, leaving only the test results from diagnostic submissions for analysis.

Data on virological tests were the results of different procedures, including real-time RT-PCR, immunohistochemistry, and virus isolation, that could be applied on different or on the same sample within the submission. Any test that indicated a positive result for influenza virus or type A influenza virus, or a specific subtype of type A influenza virus was considered as a positive individual virological test. Then, the number of virological tests performed and the number of positive virological tests were determined for each submission. Findings of individual tests that indicated a result that was “suspicious” (*n* = 12) or “weak-positive” (*n* = 9) were considered as negative tests. At least one positive virological test for influenza virus, type A influenza virus, or for any influenza virus subtype was used to declare a submission as positive for influenza virus.

Data on serological tests also consisted of results by different procedures, including hemagglutination inhibition (HI) tests for H1 and H3, IDEXX H1, IDEXX H3, and IDEXX all influenza A antibody ELISA, that could be applied on different or on the same sample within a submission. Any test that indicated a positive result for exposure to type A influenza, or a specific subtype, or specific variant of influenza virus was considered as an individual test. Then, the number of serological tests performed and the number of positive serological tests were determined for each submission. For hemagglutination inhibition tests, a cutoff point of ≥1:36 was used to declare a positive test result. The cutoff point was selected with intention to have high specificity. With the same idea in mind, results that were reported as “suspicious” (*n* = 116) were defined as negative in this analysis. At least one positive serological test for exposure to type A influenza virus, or to any influenza virus subtype, or to any influenza virus variant was used to declare a submission as positive for exposure to influenza virus. In addition, exposure to a specific subtype of influenza virus was determined in an identical manner using results of serological tests that could be interpreted at the subtype level (i.e., H3- or H1-specific ELISA, and HI assays based on different H3N2 – [A/ Swine/Ontario/33853/05, A/Swine/Texas/4199-2/98], or H1N1 – [A/Swine/ North Carolina/01, A/Swine/Ontario/5/81] variants). Thus, HI assays for H3N2 were based on the hemagglutinin of the cluster I (A/Swine/Texas/4199-2/98) and cluster IV (A/ Swine/Ontario/33853/05) viruses of the triple-reassortant H3N2 lineage^11^, whereas HI assays for H1N1 were based on the hemagglutinin of the classical swine H1N1 viruses.

The number of total submissions for virological and serological tests, as well as the number of positive submissions on virological and serological tests, was then aggregated to a monthly level resulting in four individual datasets consisting of the number of outcomes per month. Each dataset was converted to a continuous monthly time series consisting of the number of submissions or number of positive submissions for virological and serological submissions and analyzed separately.

### Statistical analysis

Data for all 4 outcomes were first decomposed into trend, season, and residual component using the STL (*seasonal, trend, and irregular components using loess*) algorithm in R 2·15·0. The algorithm is based on the loess smoothing^12^. The smoothing window for season was set for 7 months and for trend was 15 months. A set of ordinary Poisson and negative binomial regression models with number of monthly submissions for four outcomes were constructed. Each set consisted of a model with only a fixed effect of month, or additionally of linear, quadratic, or cubic effect of year centered to 2010. The model with the lowest Akaike information criterion (AIC) was considered to be the final fixed-effect model for a particular outcome. The fixed effect of month was then tested using a likelihood ratio test. Following this, an identical set of Poisson regression models with month as a random effect was fitted. The best model was selected on the basis of the lowest AIC. The significance of the random effect was evaluated by comparison of the random-effect model with the ordinary Poisson regression model using the likelihood ratio test.

Finally, the model identified as the best random-effect Poisson regression model was refitted in the Bayesian framework with month as a Poisson regression model and with month as a correlated random effect using spatial conditionally autoregressive (CAR) model^13^. The observed count of submissions (*O*_*ij*_) in year *i* (*i* = 2005…2012) and month *j* (*j* = 1…12) was based on a Poisson distribution with mean (*μ*_*ij*_).


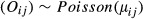



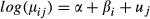


where α is the intercept, β is a the coefficient for the fixed effect of a year, where applicable, and *u* is a temporally structured random effect with variance σ^2^.

Use of spatial CAR models to assess seasonality was advocated before^14^. In the CAR model, the random effects were correlated in a sense that a specific month was adjacent to a month preceding and following the month of interest itself. Uninformative priors were used for the intercept (α∼flat), fixed linear effect of year β ∼ [Normal (0, 0·000001) when necessary, and the variance (specified as precision τ ∼ Gamma (0·5, 0·0006)]. Initial burn-in for 2 chains was based on 10E6 iterations, and an additional 5*10E6 iterations were used to produce the posterior distribution. Median, 2·5th, and 97·5th percentile were used to represent the estimate and 95% probability interval (PI) for each parameter of interest, including the random month effect. Offset was not used in these models because monthly counts were of primary interest.

To evaluate whether the proportion of positive tests within a submission showed any evidence of seasonality, random-intercept Poisson regression models were also fitted for the total number of positive samples as outcome, and number of tests as an offset. The linear, quadratic, and cubic effect of centered year was evaluated, and the best model was selected on the basis of the lowest AIC. Models with random effect of month only, and random effect of submission and month were considered. Random month effect was tested using the likelihood ratio test. Random-effect models were fitted using Gaussian quadrature method in a commercial software (stata 10.1 SE; StataCorp LP, College Station, TX, USA) or using winbugs 1.4.3 (MRC Biostatistics Unit, Cambridge, UK) for models with correlated random effects. Proportion of exposure to different subtypes was assessed descriptively.

## Results

### Monthly counts of submissions and positive submissions

Figure1 depicts the exclusion and processing of diagnostic data received from the AHL. A total of 854 diagnostic submissions were available for analysis with 659 (77·2%) requesting virological tests, 169 (19·8%) requesting only serological tests, and 26 (3%) requesting both virological and serological tests. Only 13% of submissions that had a serological test request also had a virological tests requested. Out of diagnostic samples submitted for serological assays, 33·8% had missing information about age; 0·5% was from boars, 10·3% from gilts or sows, 10·3% from finisher pigs, 2·6% from nursery or weaner pigs, and 42·6% only specified swine. For submissions made for sows/gilts, 12/20 had some history comments, with 8/12 indicating sudden outbreak of mostly respiratory disease, whereas 4/12 suggested submission of specimens from younger age-groups.

Of 2271 virological tests (Table1), ∼75% of tests were based on a type of polymerase chain reaction (PCR) test (Table1). With more than 90% of all serological tests, the subtype-specific ELISA was the most common serological test used in the study (Table1). Although the same sample could have been tested with different serological or virological tests in a sequential manner, this was not taken into account for this analysis and different tests applied to the same sample were counted as individual tests. The median number of total virological tests performed per submission was 1 (IQR = 2; min = 1, max = 91, mean = 3·1). The median number of total serological tests performed per submission was 20 (IQR = 25; min = 1, max = 120).

**Table 1:** Frequency of diagnostic tests used in the Animal Health Laboratory between 2007 and 2012

Tests for detection of antigen or nucleic acid	N	%
ELISA antigen	50	2·2
Direct fluorescent antibody test (A)	50	2·2
Immunohistochemistry (A)	391	17·2
PCR	565	24·9
Real-time PCR	1140	50·2
Typing	75	3·3
Total	2271	100·0

Tests for detection of antibodies	N	%
ELISA H1N1	2634	47·4
HI A/Swine/North Carolina/01 (H1N1)	23	0·4
HI A/Swine/Ontario/5/81 (H1N1)	54	1·0
ELISA H3N2	2538	45·7
HI A/Swine/Ontario/33853/05 (H3N2)	207	3·7
HI A/Swine/Texas/4199-2/98 (H3N2)	15	0·3
MultiS-screen type A	83	1·5
Total	5554	100·0

**Figure 2:**
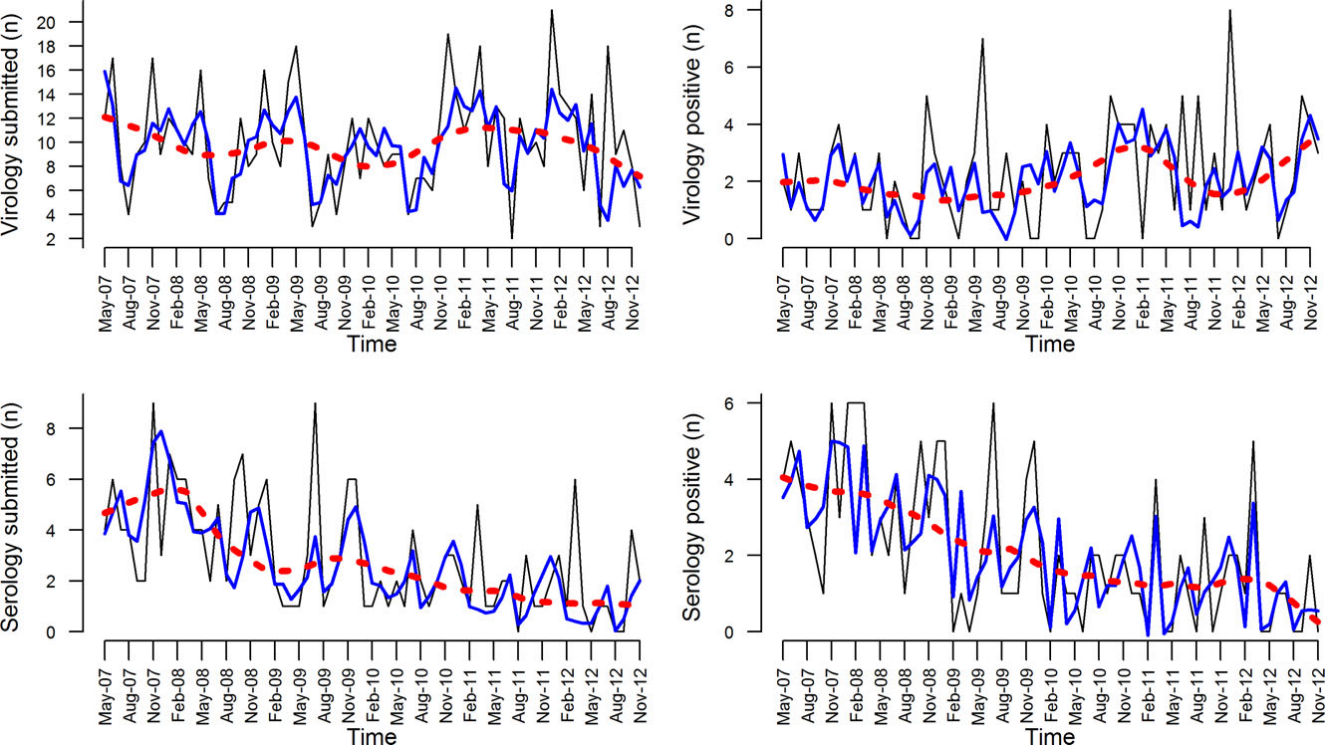
Time-series analysis. Observed data (black thin lines), smoothed trend (dashed red line), and sum of the smoothed trend and seasonal component based on the stl algorithm for 4 outcomes considered in the study of swine influenza in Ontario.

**Figure 3:**
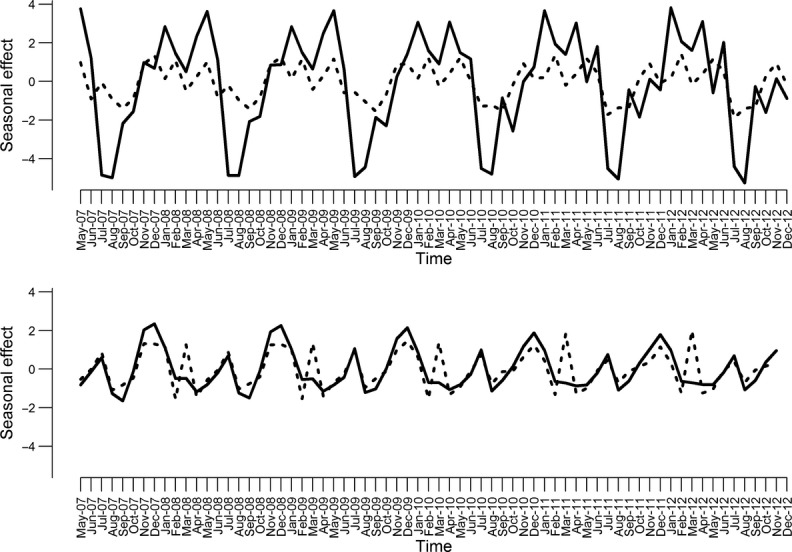
Seasonal component based on the *stl* algorithm for number of virological submissions (upper panel, solid line), number of positive virological submissions (upper panel, dashed line), number of serological submissions (lower panel, solid line), and number of positive serological submissions (lower panel, dashed line) based on submissions to the Animal Health Laboratory between 2005 and 2012.

Figure2 depicts the result of time-series analysis for all 4 outcomes performed by the *stl* algorithm and includes observed data, trend, and a cumulative effect of seasonal effect and trend. Figure3 depicts only the seasonal effect from this analysis for the submissions based on virological (upper panel) and serological (lower panel) submissions. For virological submissions, several peaks could be observed as a result of this analysis. January was the month with peak seasonal effect of virological submissions in 3 years and was the second highest in 2 years. May was the month with the peak number of virological submissions in 3 years, and April was the month with second highest number of virological submissions in 3 years. A trough in number of virological submissions typically occurred in July or August (Figure3). Based on this analysis, the seasonal effect in the number of positive virological submissions was much lower than for number of submissions, with consistently observed slight peaks in November, January, and April–May.

For serological submissions, there appears to be a first peak of submissions in the period between November and January that was consistently observed across the study period, followed by another peak in July. For the number of positive serological submissions, the pattern seemed to be congruent with the number of serological submissions, but another peak in March was consistently observed. Seasonal deviations, although consistently observed, were only moderate.

**Figure 4:**
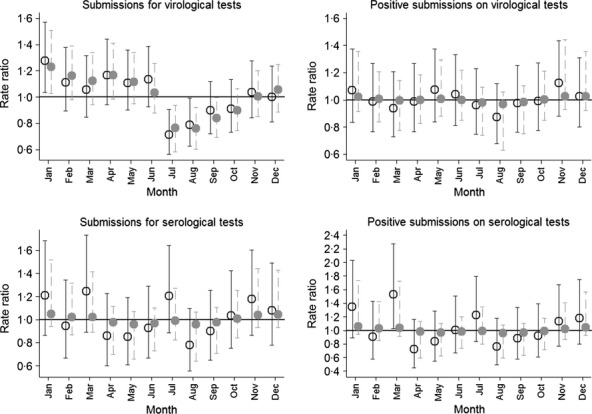
Random month effects from Poisson models with uncorrelated (hollow circles) and temporally correlated (gray circles) random effects for 4 different outcomes expressed as rate ratios where the baseline is an average month represented by a black horizontal line with origin at 1·0.

**Figure 5:**
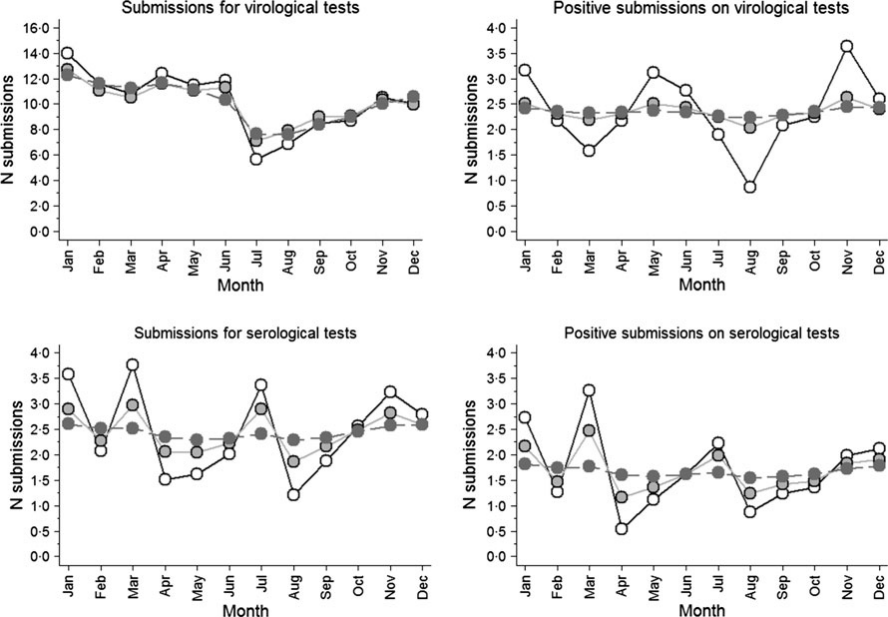
Expected monthly count of submissions for 4 different outcomes on the basis of fixed-effect model (hollow circles), random-effect Poisson model with uncorrelated random effect (light gray circles connected with solid line), and random-effect Poisson model with temporally correlated random effects (dark gray circles connected with dashed line) for the year 2010.

For the number of virological submissions, a fixed-effect negative binomial model indicated that month was associated with number of submissions (*P* = 0·011, Table2). Similarly, the random-effect model with uncorrelated random effect suggested that the random effect of month was statistically significant (*P* = 0·001). In the Bayesian model with temporally correlated random effects, the magnitude of variance was smaller in comparison with a model with uncorrelated random effects (Table2. Figure4 depicts the random effect of each month over the entire study period expressed as rate ratios in comparison with an average month (i.e., rate ratio = 1). Estimates from the random-effect model with uncorrelated random effects were expressed as estimates and 95% confidence intervals, and estimates from the Bayesian models were expressed as medians from the posterior distribution and 95% probability interval based on 2·5th and 97·5th percentile. Number of submissions was expected to be higher than in the average month in January and was expected to be lower than in an average month in July and in August. As expected, the model with temporally correlated random effects smoothed the relative risk estimates, but the seasonality pattern for the number of virological submissions was very similar between the random effect with uncorrelated and correlated random effects. The expected number of virological submissions from the fixed-effect model, random effect with uncorrelated, and random effect with correlated random effects is depicted in Figure5 (upper left panel).

**Table 2:** Final multivariable Poisson or negative binomial models for 4 different outcomes on the basis of influenza submissions data from the Animal Health Laboratory

Outcome	Covariate	Fixed-effect Poisson or negative binomial				Random Poisson with uncorrelated random effect				Random Poisson with correlated random effect		
		
		Estimate	95% CI		*P*	Estimate	95% CI		*P*	Estimate	95% PI	
N submissions for virology	Intercept	2·64	2·36	2·92	<0·01	2·30	2·17	2·43	<0·01	2·30	2·22	2·38
	Year	–	–	–	–	–	–	–	–	–	–	–
	Month*	–	–	–	0·011	0·038	0·011	0·130	<0·01	0·026	0·004	0·112
N-positive submissions on virology	Intercept	−0·14	−1·02	0·73	0·75	0·85	0·67	1·03	<0·01	0·85	0·69	1·00
	Year	0·11	0·02	0·21	0·02	0·11	0·01	0·21	0·02	0·11	0·01	0·21
	Month*	–	–	–	0·138	0·020	0·000	0·965	0·274	0·003	0·000	0·104
N submissions for serology	Intercept	0·19	−0·47	0·85	0·57	0·87	0·66	1·09	<0·01	0·89	0·71	1·05
	Year	−0·25	−0·34	−0·16	<0·01	−0·24	−0·34	−0·15	<0·01	−0·24	−0·33	−0·15
	Month*	–	–	–	0·023	0·052	0·008	0·324	0·055	0·005	0·000	0·156
N-positive submissions on serology	Intercept	−0·14	−0·90	0·61	0·71	0·48	0·20	0·76	<0·01	0·51	0·29	0·71
	Year	−0·31	−0·42	−0·20	<0·01	−0·31	−0·42	−0·20	<0·01	−0·42	−0·31	−0·20
	Month*	–	–	–	0·008	0·095	0·018	0·489	0·028	0·006	0·000	0·272

* For the fixed-effect model, month was treated as a fixed effect and tested statistically using partial likelihood ratio test. For random-effect models, month was treated as a random effect, and estimates of variance were provided. For the model with uncorrelated random effects, statistical significance was evaluated on the basis of comparison with a model with no random effect.

For number of positive virological submissions, no association between the month and the number of positive submissions could be identified (Table2, Figures4 and 5). Once correlation between the neighboring months was accounted for, the expected number of positive submissions was almost identical across all months (Figure5, right upper panel).

For number of total serological submissions, there was association between the month and the number of positive submissions based on a fixed-effect Poisson and a random-effect Poisson model (Table2, Figure4 and 5). Figure5 indicated that the highest expected number of serological submissions was in March, January, and July. However, accounting for correlation between months in the Bayesian Poisson model considerably decreased posterior distribution of between-month variance. In essence, accounting for correlation between random month effects weakened any seasonal effect that was apparent before correlation between months was accounted for.

An almost identical result to the one obtained for total serological submissions was obtained for the number of positive serological submissions (Table2, Figures4 and 5, right lower panel). In the unadjusted analysis, temporal autocorrelation for up to lag three was positive for number of virological submissions, number of serological submissions, and number of positive serological submissions, and negative (and very small) for number of positive virological submissions. Once adjusted for linear year effect, autocorrelation for number of serological submissions and number of positive serological submissions was generally negative for up to lag three (data not shown), whereas autocorrelation for virological tests remained similar to unadjusted temporal autocorrelation.

### Within-submission proportion of positive samples

**Figure 6:**
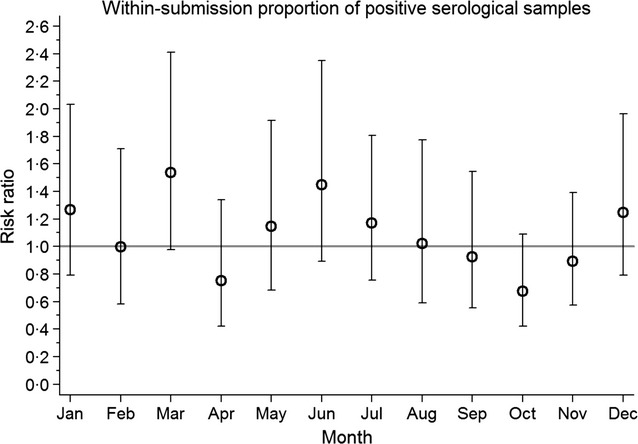
Random month effect for proportion of positive samples on serological tests. Total number of positive serological tests in a submission was used as the outcome and the total number of serological tests carried out as the exposure in this Poisson regression model with random effect of month and submission on the intercept.

The Poisson regression model that tested association between the proportion of positive virological tests within a submission indicated that the random month effect was not statistically significant, and the variance for month was almost negligible. In contrast, the Poisson regression model that tested association between the proportion of positive serological tests within a submission indicated that the random month effect (Figure6) was statistically of marginal significance (*P* = 0·06). Magnitude of variance for this model at the submission level was 1·57 (1·4–2·16), whereas the variance at the month level was 0·13 (0·02–0·65) in a model that contained centered linear effect of year. The estimates of random month effects from the latter model, together with their 95% confidence limits, are depicted in Figure6. Submissions that were submitted to the participating laboratory were expected to have the highest within-herd positivity in March, followed by June although none of them was statistically different than an average month.

### Exposure to different subtypes

**Figure 7:**
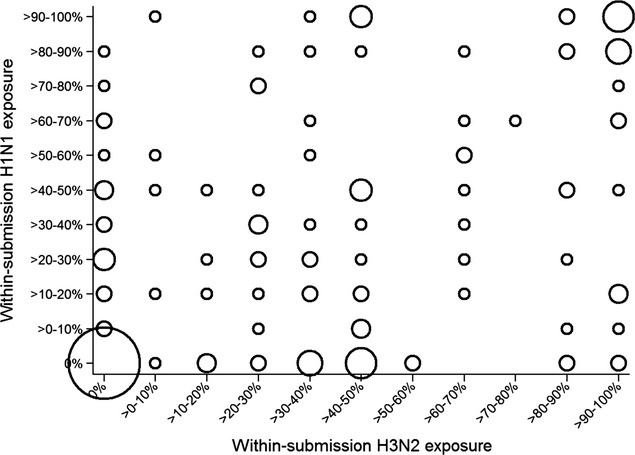
Within submission positivity for exposure to H3N2 and H1N1 influenza virus subtype on the basis of serological diagnostic data for samples submitted to the Animal Health Laboratory between 2007 and 2012.

**Figure 8:**
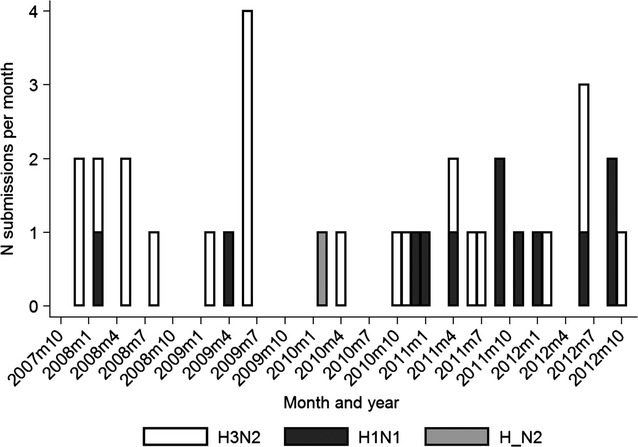
Monthly count of subtypes of influenza virus detected through the Animal Health Laboratory database between 2007 and 2012.

Table3 indicates that out of all submissions that used serological tests for exposure to H1N1 and H3N2, almost 49% of submissions were detected as positive to both subtypes. Figure7 depicts the cross-tabulation between within-submission proportion positive to H1N1 and H3N2 for submissions that were tested for exposure to the two subtypes. The within-herd prevalence was rounded to the nearest 10%. From the figure, it is apparent that there is varying degree of within-herd positivity, with multiple herds displaying high positivity for exposure to both subtypes. In 34 submissions, typing using PCR was performed. The median number of typing requested per submission was one (interquartile range = 1, mean = 2·1, maximum = 18). Ten submissions had typing requested on more than one sample. In all 10 cases, only a single subtype was detected. Of 34 submissions, 12 (35%) were detected with H1N1 subtype, 21 (62%) with H3N2, and one (3%) with N2 subtype with untyped hemagglutinin. Temporal distribution of detections is presented in Figure8.

**Table 3:** Number and proportion (%) of diagnostic serological submissions positive for exposure to H1N1 and H3N2 influenza virus subtype between 2007 and 2012.

	H3N2 −ve	H3N2 +ve	Total
H1N1 −ve	42 (25·3)	25 (15·1)	67 (40·4)
H1N1 +ve	18 (10·8)	81 (48·8)	99 (59·6)
Total	60 (36·1)	106 (63·9)	166 (100)

## Discussion

A number of unique features of herds, premises, and management are characterizing influenza virus dynamics in swine populations, and they cannot be taken explicitly into consideration in this type of analysis, although they might have large impact on transmission and seasonality. Swine demographic is very dynamic with a high birth rate that could currently reach as high as 30 pigs produced per sow per year, contributing to high inflow of susceptible animals. Contemporary swine production is based on high standards of external biosecurity aimed to limit transmission of infectious diseases between systems and flows within systems, but also allowing for planned mixing and different degree of contact within systems. Multiple subtypes or variants endemically circulate in swine, often in the same systems or herds. This could generate opportunities for reassortment, but also can elicit varying degree of cross-protection with ultimate effect on transmissibility. Finally, the unit of interest for testing and for infection control is typically a population rather than individual. All of the above could have influence on the strength of seasonality measures, which in this study was assessed on the basis of 4 outcomes.

Seasonality of diagnostic submissions aimed for detection of influenza virus was the only parameter that showed seasonality regardless of the analytical approach. This suggests existence of month-to-month variability in the level of influenza-like disease in Ontario swine herds. The pattern was characterized with peaks in January and April, then sudden decrease with a trough in July, and with subsequent steady increase in the number of submissions between July and December. Reasons for this are not clear from this analysis, but we hypothesize it could be attributed to the disease ecology itself, and to the way that disease is monitored. In temperate regions, influenza in people regularly peaks in winter months.^2^ This pattern has been attributed in part to a combination of cold temperature and particularly low absolute humidity during winter^5^ and has been supported through analysis of aggregated data from monitoring systems^15^, modeling studies^15^, and mechanistic experimental models.^16^,^17^ A comparable research is currently not available in swine to the best of our knowledge. The direct comparison of results of this study to human populations cannot be carried out for reasons stated in general comments. The level of interest is a herd rather than individual, which inevitably has impact on the number of cases reported and ultimately the strength of seasonality indicators. A peak in April is more surprising although consistent with a recent report from Minnesota.^10^ Due to indoor housing of pigs, the impact of external environment on animals is mediated through building design and ventilation system. Ventilation systems in different types of swine barns could have differing capacities to maintain the recommended microclimate under varying external conditions. Months such as April are expected to have variability in external temperatures, which could in turn influence the microclimate in barns and thereby influence the transmission of influenza virus, or clinical expression of influenza. Nonetheless, these same environmental conditions could also influence transmissibility and morbidity due to other infections with respiratory involvement in pigs, and those are numerous. Examples of such infectious agents that have been endemic during the study period in the source population are porcine reproductive and respiratory syndrome virus (PRRSV), *Mycoplasma hyopneumoniae*, and porcine circovirus type two. Although clinical diseases due to the latter two infectious agents are generally controlled through effective vaccines at present time, production issues that would prompt diagnostic submissions have been frequent during the study period. Thus, some of the patterns that have been seen in this study could also be due to cases with initially incorrect clinical diagnosis, or as a part of submissions that requested tests for multiple respiratory pathogens at the same time. Some behavioral factors may also contribute, although likely inconsequentially, to this pattern due to vacation times during summer, winter holidays in December, and possible fieldwork in spring and fall. Interestingly, the pattern for the number of virological submissions in this study was in partial agreement with an observed pattern of virologically positive submissions in Minnesota.^10^ In the latter study, a spring peak (April) was observed as well, but the highest peak of positive submissions was in the fall (October) and relatively low number of submissions in January.

In contrast, no seasonality in the number of positive virological submissions per month could be identified in this study population. This is in agreement with a general statement that seasonality of infection has been lost with the advent of modern production systems.^7^ However, it also must be pointed out that expected and observed values for the number of positive submissions were generally low, and perhaps not sufficient to detect differences between months. A seasonal increase based on 2 years of data was detected in Minnesota^10^, but the number of virologically positive submissions was considerably higher than in this study. It is worth noting that a descriptive pattern based on the *stl* algorithm, as well as fixed-effect model Poisson model, also suggested a peak in November, which agrees with the findings of the Minnesota study. Exclusion of data from certain years had an impact on the peak month when the spring increase in submissions of virological tests was found. Nonetheless, such models, based on a subset of data, also predicted an increase in virological submissions between April and June. Similarly, exclusion of certain years did not influence general predictions for virus-positive samples, but had an impact on whether this effect was found to be statistically significant. Thus, studies from multiple laboratories servicing similar study regions, and preferably over a longer period of time, should be considered for similar future investigations.

In principle, seasonality of influenza virus circulation cannot be inferred on the basis of results from serological assays, without additional demographic and clinical information. This is because a serologically positive response, measured at one point in time, is a result of exposure to influenza virus at an unknown point in the past. Longevity of antibodies also depends on the type of antibodies, the type of serological assay used, and probably on the age of animals, among other things. Examination of comments made at the time of submission suggested that submitted serological samples were obtained from a variety of age-groups and scenarios, but did not provide clear answers about the most typical scenario that prompted submission of specimens. Nonetheless, we provided results of the same analysis for serological assays for comparison purposes.

Discordant conclusions could be reached about seasonality of serological exposures on the basis of different data analysis approaches. In analysis that assumed independence of months, some evidence of seasonality existed, whereas in the analysis that assumed that months were correlated, no evidence of seasonality could be detected. A technical explanation of this issue is in the nature of temporal autocorrelation. Once adjusted for the yearly trend, the correlation up to lag three was negative. This negative correlation between adjacent months was taken into account in Bayesian models with correlated random effects, and likely contributed to extensive smoothing that resulted in absence of seasonality in these models. In general, monitoring data based on serological assays are less accurate in identifying the timing of exposure to influenza virus than data based on virological tests because there could be a considerable time lag between the exposure to the virus and submission to a diagnostic laboratory. The results of this study are likely influenced by this, because only 13% of serological submissions requested virological test for influenza at the time submission was carried out, which may suggest that majority of serological submissions were made at a time when virus was not expected to be found.

A very similar seasonal pattern, with second peak in June rather than in July, was also present for within-submission seroprevalence of influenza. Despite its marginal statistical significance, existence of this pattern warrants further investigations to better understand seasonality of influenza infections within swine farms. A previous serological study could not detect any seasonality in proportion of serologically positive pigs or herds^8^, when sampling was stratified on the basis of two broadly defined seasonal periods (winter and summer).

An interesting contrast to this was the absence of any evidence of seasonality in within-submission positivity on virological submissions. This can be explained by several distinct characteristics of samples submitted for virological influenza testing in pigs under field conditions: (i) risk-based sampling can be easily utilized during the acute phase of illness by targeting individuals and populations within an affected herd expressing typical clinical signs, (ii) pooling of individual samples is frequently practiced to increase diagnostic or surveillance sensitivity and to decrease cost of testing. The consequence of both of these approaches is to have high, or at least consistent, submission-level sensitivity that should not change between months.

A considerable proportion of diagnostic submissions in this study had samples from animals that were exposed to both influenza virus subtypes, which is in line with findings in other studies.^8^ Nonetheless, this result has to be interpreted with some caution because the majority of test results were based on subtype-specific ELISA, and because the timing of exposure to different subtypes was unknown. Previous work demonstrated that subtype-specific ELISA generally does not detect antibodies elicited with variants of another subtype.^18^ Nonetheless, exposure to H1N2 influenza virus was found to induce some cross-reactivity on H3N2-specific ELISA in the same report.^18^ Thus, possibility of cross-reactivity between different subtypes in the current study cannot be completely excluded. Nonetheless, a considerable proportion of herds had high exposure on both ELISA tests, which is arguably less likely due to cross-reactivity but to exposure to different subtypes. If two subtypes circulate concurrently in swine populations, this could result in co-infection with different viruses in the same animal and therefore create necessary conditions for reassortment to occur. Evidence of exposure of herds to the two influenza virus subtypes seemed to be consistent since the start of the study period in May 2007, which was 2 years after emergence of the triple-reassortant H3N2 virus (trH3N2) in Ontario.^19^,^11^ However, timing of exposure to different subtypes could not be elucidated. Interestingly, in a limited number of subtyping performed for diagnostic purposes, only viruses of identical subtype within the same submission were identified. This could either be due to low number of typing requests, or due to different times when exposure occurs. Previous studies identified various patterns of influenza virus circulation in a herd^20^–^22^, some of which could be contributed to the herd type, mixing patterns, or possibly circulation of drifted variants of the same subtype with unknown cross-reactivity. More detailed longitudinal studies are needed to understand cocirculation of different influenza viruses within swine herds.

A number of limitations exist in this study. It is based on passive monitoring data, which in itself could be biased and driven by different behavioral factors that could not be taken into consideration during the analysis stage. Any change in sampling or in testing strategies could have influenced the results of the study. Similarly, any outbreak or epidemic of novel influenza viruses could also influence the results of this study. The study is ecological in nature, and seasonally forced transmission has to be investigated further within specific herds over a prolonged period of time.

In conclusion, the seasonality of submissions with peak in January and April, and trough in July were detected, whereas significant seasonality of positive virological findings which should be most indicative of seasonal circulation of influenza virus could not be detected. Contradictory findings were found with respect to seasonality of serological submissions, depending on whether correlation between months was taken into consideration. Almost 50% of diagnostic serological submissions had evidence of exposure to two common influenza virus subtypes, H3N2 and H1N1.
